# Antibiofouling Activity of Graphene Materials and Graphene-Based Antimicrobial Coatings

**DOI:** 10.3390/microorganisms9091839

**Published:** 2021-08-30

**Authors:** Anna D. Staneva, Dimitar K. Dimitrov, Dilyana N. Gospodinova, Todorka G. Vladkova

**Affiliations:** 1 Laboratory for Advanced Materials Research (LAMAR), University of Chemical Technology and Metallurgy, 8 Kliment Ohridski Blvd, 1756 Sofia, Bulgaria; ani_sta@mail.bg (A.D.S.); dimitrovdimithar@gmail.com (D.K.D.); 2 Faculty of Electrical Engineering, Technical University-Sofia, 8 Kliment Ohridski Blvd, 1756 Sofia, Bulgaria; dilianang@tu-sofia.bg

**Keywords:** graphene nanomaterials, biofilms, antimicrobial coatings, antimicrobial mechanisms, bioactivity influencing characteristics

## Abstract

Microbial adhesion and biofilm formation is a common, nondesirable phenomenon at any living or nonliving material surface in contact with microbial species. Despite the enormous efforts made so far, the protection of material surfaces against microbial adhesion and biofilm formation remains a significant challenge. Deposition of antimicrobial coatings is one approach to mitigate the problem. Examples of such are those based on heparin, cationic polymers, antimicrobial peptides, drug-delivering systems, and other coatings, each one with its advantages and shortcomings. The increasing microbial resistance to the conventional antimicrobial treatments leads to an increasing necessity for new antimicrobial agents, among which is a variety of carbon nanomaterials. The current review paper presents the last 5 years’ progress in the development of graphene antimicrobial materials and graphene-based antimicrobial coatings that are among the most studied. Brief information about the significance of the biofouling, as well as the general mode of development and composition of microbial biofilms, are included. Preparation, antibacterial activity, and bactericidal mechanisms of new graphene materials, deposition techniques, characterization, and parameters influencing the biological activity of graphene-based coatings are focused upon. It is expected that this review will raise some ideas for perfecting the composition, structure, antimicrobial activity, and deposition techniques of graphene materials and coatings in order to provide better antimicrobial protection of medical devices.

## 1. Introduction

Medical device-associated infections (MDAIs) due to microbial attachment and biofilm formation on their surfaces are a persistent worldwide spreading problem with a high economic cost and impact on human health. Every day, approximately one in 31 hospital patients has at least one healthcare-associated infection [1]. Most of them are associated with medical devices such as ventilators, central lines, urinary catheters, indwelling orthopedic devices, dentistry materials, prostheses, etc. [1,2]. The MDAIs result in prolonged hospital stays, long-term disability, and a high financial cost for the healthcare systems and patients, as well as excess death [3,4]. More than 4 million patients are affected by healthcare-associated infections (HCAIs) every year in Europe, causing 16 million extra days in hospital and leading to approximately EUR 7 billion in direct costs, as reported by World Health Organization (WHO) [5]. Due to the increasing microbial pathogens resistance to one or multiple antibiotics, conventional antibiotic therapies become less and less effective. According to data presented by the National Healthcare Safety Network (NHSN) for patients within a hospital [2,6,7,8,9], each year more than 2.8 million antibiotic-resistant infections occur in the United States alone, and more than 35,000 people die as a result.

Despite the extreme efforts made so far, a total prevention of biofilm formation has not been achieved. Reduction, to some extent, was always reported. The protection of material surfaces against microbial adhesion and biofilm formation remains a significant current challenge. One general approach to mitigate the problem is surface modification or antimicrobial coating deposition to create heparinized, drug-delivering, cationic polymer or antimicrobial peptide immobilized antimicrobial surfaces and others, each one with its own advantages and shortcomings [10]. Due to increasing microbial resistance to conventional antibiotics, interest in material surface engineering by novel antimicrobial agents, and especially of carbon materials, has increased [11]. Graphene (Gr), which was discovered in 2004, is one of them. Because of its specific structure and outstanding properties, it is largely studied as a nanomaterial for potential application in different fields, such as electronics, energy storage, sensors, and many others. During the last decade, graphene-based nanomaterials have emerged as new, green, broad-spectrum antimicrobial agents for the development of drug-delivery systems, antimicrobial biomaterials and coatings, etc. [12]. The aim of this short review is to present the progress, during the last 5 years, in the development of new antimicrobial coatings based on the biological activity of graphene nanomaterials, with the expectation of raising some ideas for perfecting their composition, structure, antimicrobial activity, and deposition techniques that could provide better antimicrobial protection of medical devices.

## 2. Microbial Biofilms

**Figure 1:**
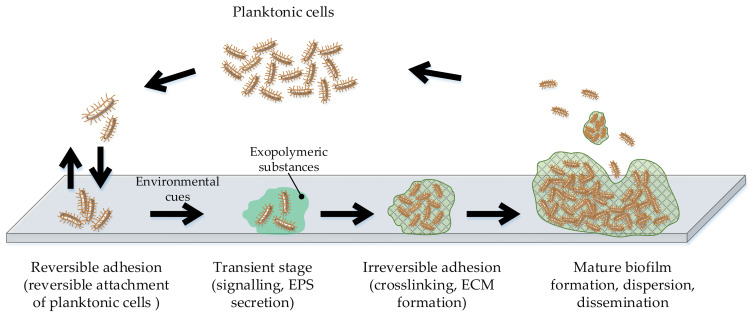
General mode of microbial biofilm formation.

Life in biofilm is the oldest, most successful, and ubiquitous form of microbial life. Biofilms are extremely capable of self-reproduction and resist traditional means of killing planktonic bacteria [13,14,15]. Knowledge about the mode of biofilm formation and its composition helps the development of antibiofilm strategies. The generally accepted mode of biofilm development (Figure 1) includes several stages: Initially, planktonic cells reversibly attach to the surface (reversible adhesion) and remain in this transition state until signaled by an environmental cue to form a less ephemeral relationship; once microorganisms begin to secrete exopolymeric substances (EPSs), biofilm develops in an irreversible process due to a cross-linking and extracellular matrix (ECM) formation, and the formed mature biofilm is capable of dispersion and dissemination [16,17,18].

In the mature biofilm, the cells are already wrapped in an extracellular matrix (ECM), composed of proteins, exopolysaccharides, and extracellular DNA (eDNA). The matrix traps nutrients, various biologically active molecules, such as cell communication signals, and enzymes that are able to degrade various matrix components, any nutrients, and other substrates [15]. Any stage of a biofilm development can be a target of an antibiofilm treatment, but it is generally accepted that the combat with the biofilm is the easiest at the initial stage, i.e., during the reversible attachment (reversal adhesion) of planktonic microbial cells. The combat with the biofilms is complicated by several phenomena: the secretion of different EPSs by different microbial species, as well as by one and the same microbial cells on different surfaces; the versatile nature of adhesive proteins using different adsorption mechanisms in front of complementary surfaces; the concurrent adsorption of EPSs constituents, similar to the Vroman effect in the adsorption of mixed proteins [19], etc. The adhesion of microbial cells to the material surface is always the event, initiating each step in the development of biofilm, and therefore, if one is able to stop this process, the development of biofilm will be prevented [11,16,18].

A large variety of approaches and a number of materials are in use for development of antimicrobial surfaces and coatings: strong hydrophilic and strong hydrophobic polymers and polymer gels, antimicrobial peptides, specific drugs and biodegradables, new antibiotics, nanostructured composite coatings, metal and metal oxide nanoparticles, enzymes, quorum sensing (QS) inhibitors, antiadhesion agents, bacteriophages, etc., all of which are considered as experimental [10,11,20,21,22,23].

A challenge for the surface engineering of antibiofilm material surfaces is to prevent the adhesion or to kill microorganisms and biofilm formation, which could be achieved by a variety of approaches, among which is the formation of a suitable nanomaterial layer or coating, repulsing or killing the fouling organisms. Lately, because of their antimicrobial activity, graphene nanomaterials are largely studied in such treatments [22,24].

## 3. Antimicrobial Activity and Applications of Graphene Nanomaterials

The new developed 2D nanomaterials are one more basis for production of effective broad-spectrum antimicrobial agents and antimicrobial coatings on their basis. In most cases, the new graphene-based materials are nanocomposites, containing biologically active metal or metal oxide nanoparticles (NPs) and polymers with expected synergetic effects [12,25].

### 3.1. Graphene and Its Derivatives

Three main types of graphene materials: graphene (Gr), graphene oxide (GO), and reduced graphene oxide (RGO) are in use for the development of new antimicrobial agents. Their antimicrobial activity depends significantly on the method of synthesis, which determines the sheet number and thickness, as well as the oxygen content [26,27].

Systematically studying the antibacterial capacity of GO in both macrophages and animal models, Wu et al. [28] showed, in vitro and in vivo, that GO is an efficient antimicrobial nanomaterial against multidrug-resistant bacteria. Three types of bacteria capable of producing biofilms, *Klebsiella pneumonia* (*K. pneumonia*), *Escherichia coli* (*E. coli*), and *Pseudomonas aeruginosa* (*P. aeruginosa*), were used for in vitro study. *K. pneumonia* was used also as an example of a multidrug-resistant (MDR) bacterium for in vivo study by introducing GO intranasally into mouse lungs. It was found that GO can prohibit the growth and spread of *K. pneumonia* both in vitro and in vivo, resulting in a significantly increased cell survival rate, less tissue injury, subdued inflammatory response, and prolonged mice survival. The ability of GO to combat multidrug-resistant bacteria is the focus of many studies, together with different surface modifications and functionalization with inorganic nanostructures, biomolecules, and polymers as a way to reduce the toxicity and to increase the GO efficiency as an antimicrobial agent [29]. Bregnocchi et al. [30] investigated the possibility of using Gr NPs as filler of dental adhesives as a nontoxic hydrophobic nanomaterial with antimicrobial and antibiofilm properties. A significantly lower vitality of *Streptococcus mutans* (*S. mutans)* cells was demonstrated in contact with the Gr NP-filled dental adhesives. Biofilm growth on dentine tissues, covered by the adhesive, demonstrated antiadhesion properties. We found a good antifungal potential against *Candida lusitaniae* [31] and a broad-spectrum antibacterial activity [32] in porous collagen composites of RGO, synthesized according to the method of Hammer, modified by us. Giulio et al. [33] investigated in vitro the antimicrobial and antibiofilm efficacy of GO against chronic wounds pathogens: *Staphylococcus aureus* (*S. aureus*), *P. aeruginosa*, and *Candida albicans* (*C. albicans*) clinical isolates. A significant inhibition of the biofilm formation and a reduction of mature biofilm were recorded for each detected microorganism. Xia et al. [34] discussed GO and RGO as new, promising biologically active agents for antibiotic independent antibacterial applications. Due to their specific structure and physicochemical properties, GO and RGO hold great potential for development of drug-delivery systems, in photodynamic/photothermal therapy and in antimicrobial protective coatings for medical devices. Valentini et al. [35] prepared two different graphene oxides, one chemically synthesized and the other one electrochemically synthesized, characterized them (using AFM, Raman and FTIR spectroscopies, XPS, and TG/DTA), and studied their antibacterial properties (by spectrophotometer and viable cell count) using Gram-negative *E. coli* and Gram-positive *S. aureus*. The results demonstrate that, compared to the chemically synthetized GO, the electrochemically synthetized one exhibits a significantly higher bacteriostatic effect on both pathogens.

### 3.2. Graphene-Based Nanocomposites

The development of Gr-based nanocomposites aims at the improvement of their broad-spectrum antimicrobial activity due to synergistic effects. Table 1 summarizes recently reported [36] nanocomposites with improved biological activity: graphene/Ag NPs containing other antibacterial nanoparticles, polymers, or enzymatic bactericides.

Multifunctional, recyclable, synergistic nanocomposites with high efficiency toward Gram-negative bacterium, *E. coli*, and Gram-positive bacterium, *S. aureus*, were prepared by growing both iron oxide nanoparticles (NPs) and silver nanoparticles (Ag NPs) on the surface of graphene oxide GO [37]. Several types of RGO-based nanocomposites were synthesized according to the method of Hummer, modified by us: RGO/Ag [50], RGO/Ag/Cu [51], and RGO/ZnO/TiO2/SiO2 [52] demonstrated increased antimicrobial activity, compared to RGO. Gr/cadmium sulfide nanocomposites, obtained by two-step solve-thermal process, were reported with experimentally demonstrated inactivation performance toward *E. coli* in the presence of humic acid, under visible light irradiation [38]. Synergic bactericidal effect of RGO/Ag NPs nanocomposite was reported against human pathogenic multidrug-resistant bacteria: Gram-positive, *S. aureus*, and Gram-negative, *E. coli* and *Proteus mirabilis* (*P. mirabilis*). RGO/Ag NPs nanocomposite was more effective against all three pathogens than either RGO or Ag NPs. Compared to the antibiotic nitrofurantoin, this nanocomposite was equally active against *P. mirabilis* and *S. aureus*, and more effective against *E. coli*. The bacterial inhibition by RGO/Ag NPs nanocomposite was faster than that by nitrofurantoin [39]. Hyaluronic acid (HA)-template Ag NPs/GO composites for therapy of bacterial infections were developed by Ran et al. [40]. These nanocomposites provide antibacterial activity against *S. aureus* combined with low toxicity to mammal cells. In addition, they show excellent in vivo antibacterial properties in a wound disinfection model. Other Ag NPs/RGO composites (Ag NPs diameter of 16 ± 3.7 nm) prepared by coprecipitation were reported to effectively inhibit the development of Gram-negative and Gram-positive bacteria such as *S. aureus*, *E. coli*, and *P. aeruginosa* [41]. A facile synthesis method was presented for the fabrication of uniform silver oxide (Ag_2_O) decorated GO nanocomposite (Ag_2_O/GO) using sonication. A comparative study of the antibacterial properties of Ag_2_O, GO, and Ag_2_O/GO nanocomposite (by diffusion assay, colony forming ability, and cell membrane permeability) using drug-resistant Gram-negative *E. coli*, *P. aeruginosa*, and *K. pneumoniae* and Gram-positive *S. aureus* demonstrates a dose-dependent inhibition of the biofilm formation [42]. Huong et al. [43] tried to optimize the antibacterial activity of Ag NPs decorated GO nanocomposites, using glucose as an ecofriendly reducing agent in an in situ method to turn Ag+ into Ag NPs. Uniformly distributed Ag NPs (with average size of 17.68 ± 4.48 nm) onto GO sheets were found by FTIR, X-ray diffraction, Raman spectroscopy, SEM/EDX, and XPS. Higher antibacterial activity of the Ag NPs/GO than that of the precursors (Ag NPs and GO) was found against *S. aureus* and *Salmonella enterica* (*S. enterica*) by optical density and plate colony counting.

GO contains a poly(aromatic) structure and a variety of oxygen functional groups, which can form π-type metal ion−aromatic or metal ion−oxygen interactions with transition metals. This makes GO a promising dispersant and carrier of Ag NPs. Thus, Ag NPs/RGO nanocomposites were fabricated without additional dispersing agent, and have excellent antibacterial activity towards both Gram-positive *S. aureus* and Gram-negative *E. coli* [44]. Martini et al. [45] studied the antimicrobial and antibiofilm properties of GO coating on dentin surface, evaluating, in vitro (by the colony forming unit counting method), its ability to prevent the *Enterococcus faecalis* (*E. faecalis*) adhesion. The GO on dentin discs demonstrated high antibacterial activity. The GO film demonstrated acceptable adhesion to root dentin, a significant inhibition of the bacterial proliferation and biofilm formation. Water-soluble Ag/GO nanomaterials were synthesized by Zhu et al. [46] under ultrasound-assisted conditions and characterized by FTIR, X-ray diffraction, TEM, and SEM/EDX. The results showed that the silver particles are strongly attached to the GO surface and that the Ag/GO composites could inhibit the growth of *S. aureus*. A one-step route to Ag/GO nanocomposite formation, excluding the need for surfactants and reductants, was reported and a high antibacterial activity of the as-prepared Ag/GO nanocomposite toward *E coli* was found [47]. Synthesis, characterization, and antibacterial activity of Ag NPs decorated GO nanocomposite, fabricated by coprecipitation with green reducing agent, were reported also [48]. Selective antibacterial activity, significantly stronger than that of RGO itself, without toxicity to mammalian cells, was reported by Tu et al. [49] for functionalization by copper ions RGO. The copper ions on RGO are positively charged and strongly interact with negatively charged bacterial cells to achieve antibacterial activity. RGO not only actuates rapid delivery of copper ions and massive assembly onto bacterial cells, but also leads to a shift of the copper ions valence from Cu^2+^ into Cu^+^, which greatly enhances the antibacterial activity. Cao et al. [53] reviewed the antibacterial and antibiofilm abilities of graphene and its derivatives in solution and on the surface, as well as their toxicity and possible mechanisms.

### 3.3. Potential Applications of Graphene Nanomaterials

**Figure 2:**
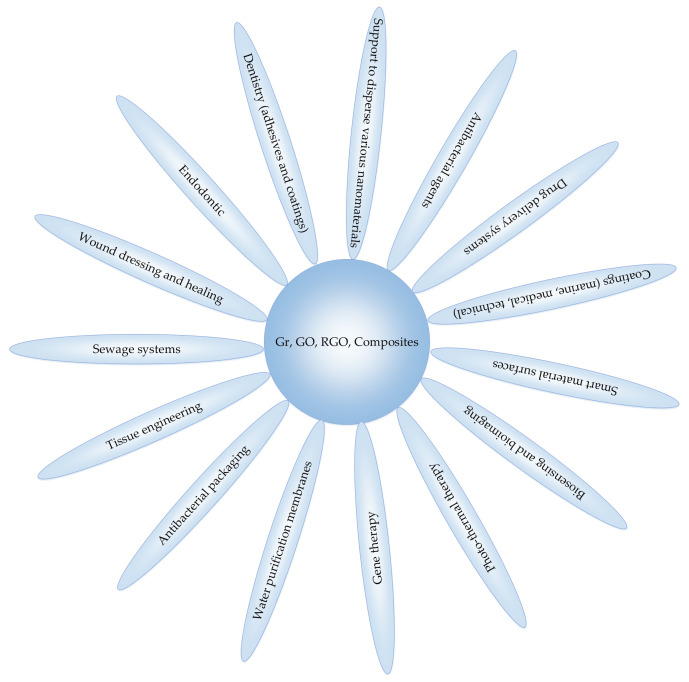
Potential antimicrobial applications of graphene, graphene derivatives, and composites.

The outstanding physicochemical characteristics, antimicrobial activity, and biocompatibility of graphene, its derivatives, and nanocomposites make them promising candidates for a large variety of antimicrobial applications, presented in Figure 2. They could be summarized as follows [54,55,56]: support to disperse and stabilize various nanomaterials, such as metals, metal oxides, and polymers with high antibacterial efficiency due to the synergistic effect [55]; antibacterial agents for treatment of multidrug-resistant bacterial infections [34,57]; drug-delivery systems (based on the two-dimensional planar structure, large surface area, chemical and mechanical stability, and good biocompatibility) [34,58]; coatings for medical devices, membranes, and others, due to bread-spectrum antimicrobial activity [59,60,61,62,63,64]; creation of smart material surfaces (graphene materials with controllable wettability) [65]; biosensing and bioimaging (due to the ability to conjugate biomolecules and fluorescent dyes) [54], photothermal therapy (because of the high near-infrared absorbance of the graphene) and gene therapy [54]; dentistry adhesives and dentin coatings [30,45]; endodontic (irrigants and intracanal medicaments; root canal disinfection) and the regenerative endodontics (support of bioactive molecules and enhancing the scaffold properties [66]; wound dressing and healing [33,40,67,68,69,70,71]; sewage systems [72]; tissue repair, tissue and organ engineering (made possible by the ability of Gr materials to stimulate the growth of eukaryotic cells and to inhibit the microbial cells attachment and growth; 3D printing of 2D graphene to fabricate 3D structure for bone tissue scaffolds) [54]; antibacterial packaging [73]; water purification membranes [74].

## 4. Antimicrobial Coatings Based on Graphene Materials

Biofilm formation on the surface of medical devices causes heavy medical device-associated infections. Deposition of antibiofilm coatings is one of the most popular approaches to mitigation the problem. A variety of coatings for antimicrobial protection of medical and other devices has been developed so far [75,76,77,78,79,80,81,82,83,84,85,86,87,88,89,90,91,92,93,94]. Unfortunately, no one was able to completely prevent the biofilm development, and the search for new solutions continues. The progress in the synthesis of graphene-based nanomaterials and composites with unique properties and broad-spectrum antimicrobial activity creates additional opportunities for the development of new, more effective, antimicrobial coatings. They are based on graphene (Gr) and its derivatives: graphene oxide (GO) and reduced graphene oxide (RGO) or Gr composites. The research in this field is at the beginning, but it is rapidly progressing [95]. A number of papers describe deposition of graphene coatings by different methods, such as those of printed electronic, chemical vapor deposition (CVD), dipping, spraying, spin, bar, or electrophoretic coating, etc., each one with its advantages and limitations. Many composite coatings, aimed especially at improvement of the antimicrobial activity, are currently in development. Table 2 presents a number of Gr, Gr derivatives (GO, RGO), and Gr composites-based antimicrobial coatings.

### 4.1. Coatings Based on Graphene and Graphene Derivatives (GO, RGO)

Thin, horizontally grown Gr coatings were produced by chemical vapor deposition (CVD) [76]. Vertically grown Gr layers were produced by plasma-enhanced chemical vapor deposition (PECVD) [77] by placing the sample in a vacuum under chamber, heating to a high temperature, and releasing of three gases (usually hydrogen, methane, and argon) into the chamber. Under the high temperature, the gas molecules react with each other, and a thin layer of carbon atoms is deposited. When an electric field (a plasma) is applied over the sample, it causes gas ionization near the surface. Thus (with the plasma), the carbon grows vertically from the surface, forming a thin layer (instead of horizontally with CVD). Wei et al. [85] investigated the antimicrobial properties of vertically and horizontally aligned graphene, grown on a semiconductor silicon (Si) and the insulator silicon dioxide (SiO_2_). They reported different antibacterial activity against Gram-positive (*S. aureus* and *S. epidermidis*) and Gram-negative bacteria (*E. coli* and *S. typhimurium*) and explained the reasons. Zhou et al. [82] presented an ultrafast method for direct growth of uniform graphene on a SiO_2_/Si substrate using methanol as a carbon source. The high growth rate is attributed to the quick pyrolysis of the methanol with the help of copper atom traces. The as-grown graphene exhibited a high and uniform thickness, suitable for transparent conductive electrodes in electrophoretic displays applications.

Gomes et al. [78] evaluated whether surface immobilization of Gr nanoplatelets (Gr NPLs) provides antimicrobial properties to silicone rubber (SR) catheters. Gr NPLs or their oxidized form (GO NPLs) were immobilized on the silicone surface from a corresponding dispersion by dip and spray coating. The antimicrobial effect was assessed against *S. epidermidis*. Independently of the deposition technique, GO NPLs coatings induce higher bacterial death. Dipping SR/GO NPLs coatings were the most promising approach, preserving bacterial adhesion on the level of silicone while increasing bacterial death to approximately 80%. Dubey et al. [79] prepared an atom-thick Gr coating on medical grade titanium that promotes osteoblast maturation and inhibits biofilm formation from a variety of microbial species. To avoid the disadvantages of the wet transfer (which employs hazardous chemicals, limiting clinical applications), a dry transfer technique was developed, based on a hot-pressing method. It allows coating of titanium substrates area, >90%, with high-quality graphene, in a single transfer. The graphene-coated titanium is biocompatible and does not induce cell membrane damage; it induces human osteoblast maturation (at gene and protein level) and increases the deposition of mineralized matrix. In addition, Gr decreases the formation of biofilms from *S. mutans* and *E. faecalis* without killing the bacteria. Muthu et al. [80] prepared hydrophobic, bacteria-repellant Gr coatings on hydrophilic glass surfaces, recycled from pencil tubs by sonication exfoliation technology. A repellence of bacteria was demonstrated against four different biofilm-forming pathogens. Song et al. [81] evaluated the influence of GO on biofilm formation, using *E. coli* and *Bacillus subtilis* (*B. subtilis*) as models of Gram-negative and Gram-positive bacteria. The growth profiles and viability assays indicated that GO exhibits a high antibacterial activity, reducing the bacterial growth more strongly with increase of the GO concentration. Choudhary and Das [83] reported bioreduced GO as a nanoscale antimicrobial coating for medical devices. The cell biomass of *Rhizopus oryzae* was explored as a reducing agent for ecofriendly synthesis of RGO and minimizing the extensive use of toxic chemicals. Akhtari et al. [99] assessed the performance of GO NPs in paper-coating formulations in order to improve the antibacterial, physical, and mechanical properties of a paper board. The antibacterial assay was performed using *E. coli* and *S. aureus* as Gram-negative and Gram-positive bacteria, respectively. Feng et al. [65] investigated factors that affect the wettability of Gr (defects, controllable atmosphere, doping, electric field, etc.) to evaluate its ability to serve as a coating with tunable wettability for further development of smart material surfaces. Borges et al. [84] demonstrated that the exposure of small and oxidized Gr nano plates on PU surface improves its antimicrobial performance. Jankus [86] developed an economical and environmentally friendly method for producing GO from coal in a one-pot process. A comparative study of the antimicrobial properties of graphite- and coal-derived GO as robust coatings for titanium implants demonstrated the advantages of the latter. It is an inexpensive coating providing improved bone cell adhesion and hard tissue compatibility.

### 4.2. Graphene Nanocomposite Coatings

A large variety of antimicrobial graphene nanocomposite coatings have been reported lately. Their development is aimed at the adjustment of some properties for potential specific applications. In most cases, the focus is on the improvement of the antimicrobial activity: its increase and/or broadening of the antimicrobial spectrum due to synergistic effects. In other cases, the antimicrobial activity is combined with other functions, such as antithrombogenity, osteointegration, low adhesion for nontoxic control of biofouling, etc. For example, graphene-based porous coatings with antibacterial and antithrombogenic function for cardiovascular therapy were developed by introducing RGO flakes into the porous structure of the polyelectrolyte-based coatings, deposited using layer-by-layer method. Reduced bacterial film formation was verified using *E coli* and *Staphylococcus* bacteria. Blood−material interaction was examined in dynamic flow conditions. Bacteriological analysis shows reduced presence of bacteria after contact with the graphene flake-containing surface [87]. Antibacterial silver/hydroxyapatite/graphene (Ag/HAP/Gr) composite was electrophoretically deposited on medical-grade titanium to assemble a homogenous coating with improved stability in simulated body fluid (SBF) [88]. Gr and Ag NPs decorated Gr nanolayers were prepared by spray coating, and their ability to prevent the formation of *S. epidermidis* biofilm on the surface of a Foley catheter was demonstrated [89]. Chitosan cross-linked GO nanocomposite films for food packaging were reported by Grande et al. [73]. Slate et al. [75] discussed the antimicrobial efficacy of carbon-based nanomaterials (graphite, graphite oxide, reduced graphite oxide, Gr, GO, and RGO) with a special focus on the utilization and application of 2D carbon nanomaterials in surface coatings. A ternary nanocomposite of elastomeric silicone/GO sheets/Al_2_O_3_ hybrid nanorods was fabricated (via a two-phase method) and a superhydrophobic antifouling coating was prepared (via solution casting) by Selim et al. [90]. GO-based nanocomposites decorated with Ag NPs were developed by Jaworski et al. [91] as a novel multifunctional antibacterial and antifungal material. Ultrasonic technology was used as effective method for coating deposition on polyurethane foils. Toxicity toward Gram-negative bacterium, *E. coli*, Gram-positive bacteria, *S. aureus* and *S. epidermidis*, and pathogenic yeast (*C. albicans*) was evaluated, analyzing the cell morphology and cell membrane integrity (lactate dehydrogenase assay); the cell viability assessing (Presto Blue assay) and reactive oxygen species (ROS) production. The new nanocomposites show sharply increased antimicrobial efficiency toward bacteria and yeast cells, compared to Ag NPs and GO. Synergistic effects of Gr-based silver nanocomposites and composites with other antibacterial nanoparticles (polymeric or enzymatic bactericides) are reported by Aldhart et al. [36] to be utilized for surface modifications in the healthcare setting. RGO/TiO_2_ nanocomposite coatings for cotton fabrics with antibacterial and self-cleaning properties are reported by Stan et al. [89]. Their experiments demonstrate that the coatings inhibit *S. aureus* and *E. faecalis* growth and they are harmless for human skin cells. Jin et al. [93] described inspiration by unstable surfaces of naturally antifouling organisms, preparation of preventing biofouling, graphene/silicone rubber (Gr/SR) composite membranes with low surface energy and adjustable elastic modulus. Li et al. [94] report GO/lysozyme ultrathin films with strong antibacterial action and enhanced osteogenesis. Innovative protective coatings based on Gr films and hydrogels were discussed by Cacaci et al. [100] as an innovative solution to the prevention of nosocomial pathogens colonization on implantable devices. Zuo et al. [101] developed antibacterial chitosan (CS) hybrid films with N-halamine-functionalized-GO. As a precursor of N-halamine, 3-epoxypropyl-5,5-dimethylhydantoin (GH) was synthesized and attached onto GO for enhanced antibacterial activity. After chlorination by household bleach solution, the chlorinated GO-3-epoxypropyl-5,5-dimethylhydantoin (GO–GH–Cl) possessed great antibacterial efficacy. The as-synthesized GO–GH–Cl was added to CS solution to produce GO–GH–Cl/CS hybrid films via a solution casting. These hybrid films showed excellent antibacterial activity and could kill 100% of *S. aureus* and 100% of *E. coli* within a contact time of 10 min and 30 min, respectively. Khalili et al. [95] deposited, electrophoretically, hydroxyapatite/GO/ZrO composite coatings on titanium substrate. The thickness and uniformity of the created coating, the distribution of the nanopowder particles, the position of the materials used in the coating, and the corrosion behavior were evaluated by SEM, elemental analysis, X-ray diffraction, and electrochemical analyses, respectively. The antibacterial tests, performed with *E. coli* and *S. epidermidis*, demonstrated that the hydroxyapatite/GO/ZrO nanocomposite coatings effectively decrease the bacterial growth on the surface [95]. Enhanced anticorrosion and antibiofouling properties of graphene oxide–silica–polydimethylsiloxane (GO/Si/PDMS) coating on carbon steel were demonstrated by Balakrishnan et al [96]. Electrochemical analyses of GO/Si/PDMS-coated carbon steel exposed to Gram-positive *Bacillus* sp., Gram-negative *Pseudomonas* sp., and freshwater bacterial cultures show 3–5 orders of magnitude of reduction, compared with polished specimens. Confocal laser scanning microscopic analysis confirmed a significant reduction of biomass and biofilm thickness.

A hybrid siloxane-epoxy (SE) coating, reinforced with worm-like graphene oxide (WGO) nanoscrolls, was obtained for a protection of AA2024 alloy. GO sheets were firstly functionalized with tetraethoxysilane (TEOS) through a sol–gel method and incorporated into the SE via a wet transfer method. Chemical structure and morphological analyses reveal that −Si−OH groups guide the transformation of WGO into the tubular structure. SE/WGO coatings are ecofriendly and are characterized by high cross-linking density, toughness, flexibility, and easy production [97]. Sharif et al. [98] developed and characterized nanocomposite coatings based on modified GO and polydimethyl siloxane (PDMS). Surface modification of GO was carried out with 3-aminopropyltriethoxysilane as an amination agent, and coating compositions, based on PDMS containing GO or modified GO, were prepared. The interaction of the aminated GO with the siloxane polymer was evaluated by Fourier transform infrared spectroscopy–attenuated total reflectance (FTIR-ATR), SEM, AFM, Raman spectroscopy (RS), and X-ray diffraction (XRD). Hydrophilic/hydrophobic balance of the coated surfaces was estimated by water contact angle (WCA) measurement. Green synthesis of Gr/PDMS-based coatings, reducing the adhesion of fouling organism, was reported by Solemani et al. [61]. GO was reduced with *Avicennia marina*/Ag to achieve RGO/Ag nanocomposite (green synthesis approach). The simultaneous presence of *A. marina* and silver element in the structure of graphene-based nanocomposite showed a synergistic effect on the performance of Gr/PDMS-based coatings. Song et al. [62] reported a chemical, in situ method for synthesis of copper-decorated GO (GO/Cu) spin coating. A comparative study of the growth of *E. coli* and *S. aureus* on GO/Cu and pure GO coatings showed that the growth of both *E. coli* and *S. aureus* is significantly inhibited on the GO/Cu coating, whereas the GO coating does not affect their growth. Bone mesenchymal stem cell (BMSC) adhesion, viability, and proliferation indicated that the two GO-based coatings do not show toxicity, compared to a SiO_2_ control. Bouchareb et al. [63] prepared nanocomposite films with good mechanical properties by a solution blending of polysulfone (PSU) and different amounts (0.00–1.00 wt.%) of GO/AgNPs. Antibacterial testing showed that the as-prepared nanocomposite films have a significant bactericidal capability against both Gram-negative (*E. coli*) and Gram-positive (*S. aureus*) bacteria at very low GO/Ag NPs loading (0.2 wt.%). Pandit et al. [64] overviewed graphene-based antimicrobial biomedical surfaces, including graphite, Gr, GO, and RGO. This review focused on the biomedical devices with coatings or highly structured polymer nanocomposite surfaces of Gr derivatives for antimicrobial activity and their potential applications to prevent cross infections.

Silicone is one of the materials most often used for fabrication of medical devices; however, the silicone is a chemically inert, flexible, strongly hydrophobic, and low-adhesive material that makes the deposition of stable coatings on its surface difficult. Several techniques are proposed for coating of silicone with Gr or Gr-based materials and composites: spring (usually after preliminary surface activation), chemical vapor deposition (CVD), or plasma-enhanced chemical vapor deposition (PECVD), electro photoelectric deposition (EPD), or metal assisted exfoliation (MEA) [102,103].

### 4.3. Wound Dressing and Healing

Hydrogels with antibacterial performance and good water-maintaining ability are of interest for the development of wound dressing. Prepared by crosslinking of Ag/Gr composites with acrylic acid and *N*,*N*′-methylene bis-acrylamide was reported by Fan et al. [68], with abilities to effectively kill bacteria and to accelerate wound healing, demonstrated in vivo on artificial wounds of rats. We developed porous collagen-based materials with broad-spectrum antibacterial activity for wound dressing and healing. Synthesized by us, RGO and RGO/SiO_2_ [32], RGO/Ag, and RGO/Ag/SiO_2_ [104], as well as RGO/ZnO/TiO_2_/SiO_2_ [105], were used as new antimicrobial agents. Sol−gel cryogen drying was employed for fabrication of all composites to keep the native biological activity of the collagen. It was found that, compared to RGO, RGO/Ag and RGO/ZnO/TiO_2_ demonstrate an increased antibacterial activity to broad-spectrum Gram-negative and Gram-positive bacteria due to synergistic effects. SiO_2_ also contributes to the improved antimicrobial activity, acting as dispersant, which leads to the more homogenous distribution of the antimicrobial agent in the collagen matrix.

Used in wound dressing, bacterial cellulose (BC) has no antibacterial activity itself. Such could be added by impregnation with optimized graphene oxide silver (GO/Ag) nanohybrid. Compared to silver nanoparticles, GO/Ag nanohybrids are more effective and show synergistically enhanced antibacterial activities at low doses. The GO/Ag nanohybrids are more toxic to *E. coli* than to *S. aureus* [67]. An efficient wound dressing, based on silk fibroin (SF), polydopamine (PDA), chitosan (CS), and RGO (PDA/RGO/CS-SF) was reported by Tang et al. [69]. Briefly, inspired by mussel chemistry, a PDA/RGO was prepared under alkaline conditions and dispersed in a CS/SF mixture. CS and SF chains were cross-linked by poly(ethylene glycol)di(glycidyl) ether and glutaraldehyde to obtain a PDA−RGO-incorporated gel; a freeze-dry process was applied to obtain a PDA/RGO/CS-SF scaffold. This scaffold is able to promote physiological electrical signal transmission for cell growth and reduces ROS oxidation, resulting in an improved wound regeneration as demonstrated at in vitro testing and in vivo experiments. Yang et al. [70] developed GO-coated shell−core-structured chitosan/poly(lactic acid) (CS/PLLA) nanofibrous scaffolds for wound dressing. GO nanosheets are coated on the shell of CS/PLLA core without destroying the nanofiber structure. The successfully coated GO nanosheets on CS/PLLA nanofibrous scaffolds significantly improve their hydrophilicity and antimicrobial activity toward Gram-negative (*E. coli*) and Gram-positive (*S. aureus*) bacteria. Rat wounds covered by GO-coated CS/PLLA nanofibrous scaffolds heal better than other groups on pathological sections [70]. A potential use for wound dressing is proposed also for Ag NPs/RGO nanocomposites, fabricated without additional dispersing agent [44].

GO and stabilized ortho silicic acid were comparatively studied as modifiers of amnion and burn-affected skin. The thermogravimetric study found the highest stability of the analyzed tissues (hypo trophic amnion and burnt epidermis) after modification with graphene oxide and sodium ascorbate [68]. Giulio et al. [106] found that the GO affects *S. aureus* and *P. aeruginosa* dual species wound biofilm in the Lubbock Chronic Wound Biofilm (LCWB) model. It mimics the spatial microbial colonization into chronic wounds and reproduces the wound and its clot. The GO significantly affects both the formation and maturing of biofilms, as detected by the CFU/mg reduction, cell viability, and ultrastructural analysis. SEM images show that GO disaggregates the microbial cells, disrupting the fibrin network of the wound-like biofilm framework.

## 5. Proposed Mechanisms of Microbial Adhesion Inhibition by Graphene-Based Nanomaterials

Antimicrobial activity of Gr-based nanomaterials was demonstrated across a broad spectrum of bacteria. The understanding of the mechanisms of microbial growth inhibition by Gr nanomaterials is a key to increase their efficiency. A number of antimicrobial mechanisms and physicochemical characteristics influencing the antimicrobial activity of Gr nanomaterials are presented in the literature, including physical contact destruction; oxidative stress (ROS-dependent/independent); photoinduced antibacterial activity; controlled drug/metal ions release; synergistic antibacterial activity, etc., but they still remain not fully understood [107].

Liu et al. [108] and Krishnamoorthy et al. [109] connected the antibacterial activity of Gr materials with membrane stress and oxidative stress based on their comparative study of the graphite, graphite oxide, Gr, GO, and RGO towards *E. coli* under similar conditions. The antibacterial efficiency of graphene nanosheets, synthesized by a hydrothermal approach [106], tested against four types of pathogenic bacteria (*E. coli*, *Salmonella typhimurium*, *E. faecalis,* and *B. subtilis*) was suggested to be due to the involvement of ROS indicated by a measurement of free radical activity. Perreault et al. [110] found GO nanosheets were size dependent on the antimicrobial activity toward *E. coli* (in cell suspensions of): four-fold when GO sheet area decreases from 0.65 to 0.01 μm^2^. They supposed that in suspension assays, the GO interacts with the bacteria by a cell entrapment mechanism.

Graphene and its derivatives differ in their physical, structural (morphology: mono- and multilayer), and electronic properties and surface chemistry: Gr, GO, and RGO. Therefore, they interact differently with the microbial cells. For instance, the lateral size is important to enhance bacterial adhesion, whereas the sharp edges may act as nanoknives. GO can enhance the antimicrobial activity through oxidative stress with or without the production of ROS [111]. The main mechanisms proposed to explain the antibacterial behavior of Gr and its derivatives are grouped by Shi et al. [56] as follows: the membrane stress hypothesis; the oxidative stress hypothesis; the entrapment hypothesis; the electron transfer hypothesis; the photothermal hypothesis.

It is accepted that the Gr nanomaterials exert antibacterial action via physical and chemical damages of the bacteria. The direct contact of their sharp edges with bacterial membranes leads to a destructive extraction of lipid molecules. Such damage also includes wrapping and photothermal ablation mechanisms. The chemical damage of bacteria is caused by oxidative stress with a generation of ROS and charge transfer [55]. Szunerits and Boukherroub [112] describe the features of Gr–bacterial interactions, the importance of size and chemical composition in the inhibition of bacterial proliferation and adhesion, cytotoxicity, and other issues when considering future clinical implementation.

Rojas-Andrade et al. [113] focused on antibacterial mechanisms of Gr nanocomposite nanomaterials, prepared by combining Gr derivatives with antibacterial metal and metal oxide nanostructures that expose exceptional bactericidal activity. Zheng et al. [26] discussed the structure–activity relationship that is involved in GO-induced bacterial killing and the molecular initiating events, including redox reaction with biomolecules, mechanical destruction of membranes, and catalysis of extracellular metabolites. The most frequently proposed mechanisms of action of the Gr materials are summarized by Adlhart et al. [36] in several categories: oxidative stress induction; protein dysfunction; membrane damage; transcriptional arrest. It was demonstrated that the mechanism of action depends on the concentration of the bactericide: the low GO concentrations cut membranes of the microorganisms (*S. aureus* and *E. coli*), whereas high concentrations induce the formation of GO aggregates, shielding their edges. When cluster size increases, bacterial deactivation through wrapping is observed [110]. In efforts to find Gr nanomaterials with as high as possible biocidal activity, Palmieri et al. [114] also studied factors influencing their biocidal activity and their antimicrobial mechanisms. The particles size is presented as the most important factor affecting the antimicrobial activity of carbon materials: Gr, GO, carbon nanotubes, and fullerenes. Smaller particles with a higher surface to volume ratio can easily attach onto the microbial cells and affect their cell membrane integrity, metabolic processes, and structural components [115].

The antibacterial activities of Gr and Gr-derived materials are attributed mainly to the direct physicochemical interaction between Gr materials and bacteria that cause a deadly deterioration of cellular components, such as proteins, lipids, and nucleic acids. In fact, Gr materials hold a high affinity to the membrane proteoglycans where they are accumulated, which leads to membrane damages; similarly, after internalization, they can interact with bacterial RNA/DNA hydrogen groups, interrupting the replicative stage. Moreover, Gr materials can indirectly cause bacterial death by activating the inflammatory cascade due to active species generation after entering in the physiological environment [116].

The toxicological activity of graphene is usually related to its ability to produce ROS, which can be altered by surface modification through various transformation processes. Unfunctionalized graphene (u-Gr), carboxylated graphene (Gr–COOH), and aminated graphene (Gr–NH_2_) were selected by Yao et al. [117] to determine their ability to photogenerate ROS in the aqueous phase. Oxidative stress (ROS concentration and superoxide dismutase activity) induced by the materials was investigated. Based on density functional theory (DFT) calculations, photochemical pathways of ROS production were identified. Gr–COOH-, Gr–NH_2_-, and u-Gr-generated superoxide anions and further produced hydroxyl radicals by inducing electron transfer were detected. By comparing the biological redox potential and the lowest occupied molecular orbital values (ELUMO) of the substances, u-Gr and Gr–COOH were identified to have the potential to induce oxidative stress. The predictive results were validated by the significant increase of oxidative stress biomarkers in *Daphnia magna*. By coupling experimental observations with the theoretical predictions, the results provide mechanistic insight into understanding the photochemical activity and toxicity of graphene and its surface-functionalized derivatives. During the last decade, conflicting interactions of bacterial cells and Gr materials were reported for different potential applications. On one side, Gr materials with antibacterial activity were synthesized as an alternative to currently used antibiotics and to develop antimicrobial coatings, preventing biofilm formation on different material surfaces. On the other side, Gr materials were developed to promote the proliferation of electroactive bacteria on the surface of electrodes in bioelectrochemical systems or to accelerate interspecies electron transfer during anaerobic digestion. Gr materials were successfully employed also as proregenerative materials for tissue engineering. This raised the question of whether graphene is an antibacterial agent or a promoter of cell proliferation. To answer this, Zhang et al. [27] debated the mechanisms and factors determining the positive or negative impact of Gr materials on bacteria and summarized that adjustable physicochemical properties and environmental factors determine whether the Gr materials will act as antibacterial materials or will promote bacterial growth. The toxicity of Gr materials toward bacteria is partly explained by their capacity to cause oxidative stress by ROS generated from molecular oxygen. This simple observation raises possible concerns for the long-term stability of Gr materials in environments where oxygen is missing. Seifi et al. [57] summarized the literature, discussing various factors that affect the antibacterial performance of Gr materials, including the shape, size, functional group, and the electrical conductivity of graphene flakes, as well as the concentration, contact time, and the pH value of the graphene suspensions used in microbial tests. The possible surface and edge interactions between bacterial cells and graphene nanomaterials are discussed, which cause antibacterial effects such as membrane/oxidative/photothermal stresses, charge transfer, entrapment, and self-killing phenomena.

Yang et al. [118] designed biocompatible antibacterial materials on the base of respiratory electron transfers of bacterial cells, playing an important role in bacterial metabolism. Gr nanosheets were dispersed in biocompatible and chemically stable TiO_2_ matrix using a plasma spraying technique. It was found that the electrical conductivity of TiO_2_ coating was significantly enhanced due to combination of the unpaired π-electrons of Gr nanosheets and the Ti atoms on the surface of TiO_2_. The enhanced transfer of the extruded electrons from the bacterial cell membranes to the Gr nanosheets/TiO_2_ and subsequent electron enrichment at the Schottky-like Gr nanosheets/TiO_2_ interface leads to bactericidal action. This mechanism was validated by the documented nonantibacterial efficacy of the insulating ZrO_2_ coating doped with the same amount of Gr nanosheets, whose electrical conductivity was unchanged with the addition of Gr nanosheets and was much lower than that for Gr nanosheets/TiO_2_. Using MC3T3-E1 as a model cell, in vitro cell culture experiments proved that the proliferation and osteogenic activity of the cells cultured on TiO_2_ and Gr nanosheet/TiO_2_ coatings are comparable, indicating that the antibacterial Gr nanosheets/TiO_2_ coating possesses uncompromised cytocompatibility.

For a more in-depth understanding of the role of the ROS, GO was fabricated on a titanium surface by cathode electrophoretic deposition with and without nitrogen doping [119]. The systematically studied antibacterial activity demonstrated that GO presents antibacterial activity, while nitrogen-doped GO lost the antibacterial activity on the titanium surface. This feature is explained by two steps of antibacterial mechanisms for the GO metal system: at the first step, electron transfer occurs from the bacterium’s cell membrane to the GO surface, which destroys the bacterial respiratory chain; subsequently, electrons on the GO surface induce the production of ROSs that damage the membrane structure and lead to a possible bacterial death. For nitrogen-doped GO, nitrogen atoms donate electrons into GO, leading to n-type doping. As an electron donor, nitrogen-doped GO cuts off the electron transfer from the cell membrane to GO and subsequently inhibits the production of ROSs. In this way, the study of Qiu et al. [119] experimentally confirms the antibacterial mechanisms of GO/metal synergistic systems with an effect on nonoxidative electron transfer and ROS-mediated oxidative stress. Antibacterial and antibiofilm abilities of Gr and its derivatives in solution and on the surface were reviewed by Cao et al. [53], in the sense of some controversy as to whether graphene and its derivatives can resist infections and biofilms. The not-fully-understood antibacterial mechanisms and cytotoxicity of Gr, as well as of its derivatives, are also in the frame of this review.

**Figure 3:**
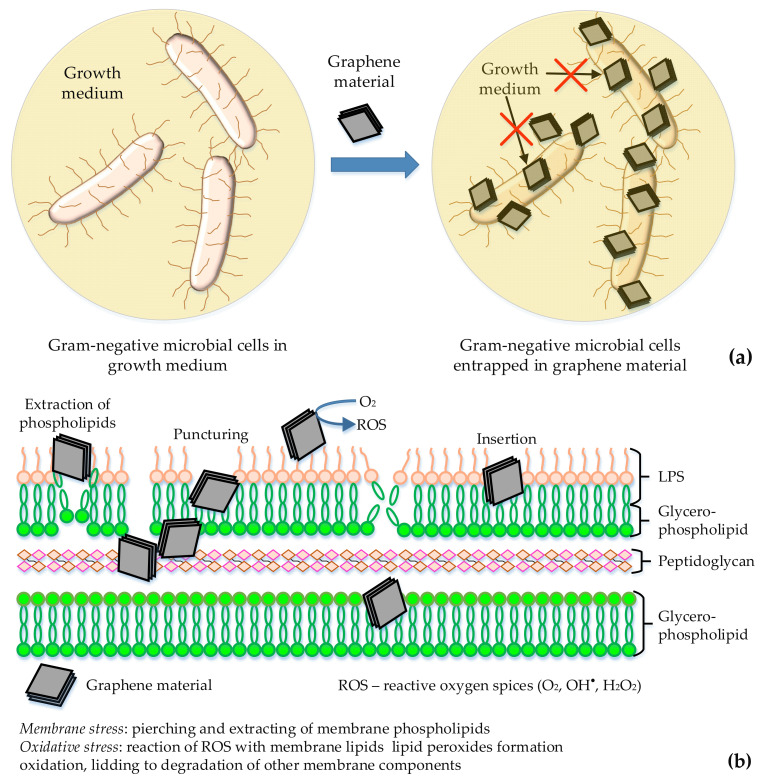
Principle sketch of bactericidal mechanism of graphene materials toward Gram-negative microbial cells: isolation of microbial cells by graphene materials wrapping (**a**); membrane and oxidative stress (**b**).

A principal sketch of the proposed bactericidal mechanism of the Gr materials is presented in Figure 3.

## 6. Factors Influencing Antimicrobial Activity of Graphene Nanomaterials-Based Coatings

The Gr nanomaterials are already accepted as promising materials for development of antimicrobial coatings; however, the study of their influence towards biofilm formation is still at the beginning. When Gr, Gr derivatives, and composites are used as a base of antimicrobial coatings, additional factors influencing their antimicrobial activity should be taken into account, such as elastic modulus, wettability of the graphene, concentration and orientation of the Gr nanoparticles on the surface, etc. [59]. Concentration-dependent antibacterial activity was found for GO on biofilm formation by *E. coli* and *B. subtilis* as models of Gram-negative and Gram-positive bacteria [81]. It is interesting that the biofilm formation was enhanced in the presence of low-dosage GO, whereas it was inhibited in the presence of high GO concentration. These results are explained by the roles of dead cells inactivated by GO. At low GO concentrations, a part of the cells is only inactivated that serves as a protection barrier but also as a nutrient to the remaining, biofilm-forming, living cells. At high GO concentrations, almost all cells can be completely inactivated, and the biofilm formation will be strongly reduced.

The importance of nanosheet surface exposure for biofouling resistance was experimentally demonstrated by Cheng et al. [74] using two strategies to prepare GO functionalized membranes: coating and blending. In contact with the model bacterium *E. coli*, the GO-coated membrane exhibits enhanced biofouling resistance as compared to the GO blended membrane. Wei et al. [85] reported different antibacterial activities of vertically and horizontally aligned graphene nanosheets, confirming the importance of nanosheets exposure for the antimicrobial properties. Other biofouling influencing factors are the elastic modulus [16,93] and the wettability [61] of the material, as experimentally found by some researchers.

Analyzing the proposed mechanisms of Gr materials’ antimicrobial action, it could be summarized that adjustable physicochemical properties and environmental factors determine whether the Gr materials will act as antibacterial material or if they will promote bacterial growth. The antimicrobial action depends on chemical composition of the carbon material (graphite, graphite oxide, Gr, GO, RGO); the size of the nanoparticles and the number of the nanosheets; the type and amount of the modifying agent (metal, metal oxide, enzyme, and others) in the Gr nanocomposites. Additional factors determining the antimicrobial performance of Gr materials-based coatings are the concentration of the Gr nanomaterial, the exposure of the Gr nanomaterial sheets to the material surface, its elasticity, and the wettability.

## 7. Concluding Remarks

The increasing microbial resistance to traditional antimicrobial treatments motivates the search for new, broad-spectrum antimicrobial agents and the development of new protective coatings on their basis. During the last years, graphene, graphene derivatives, and composites have been widely studied as antimicrobial agents for medical applications. The modification with other biologically active nanomaterials (predominantly metal nanoparticles and oxides) and combinations with polymers to obtain synergistic effect is a sustainable current trend.

No report was found regarding total prevention of biofilm formation on graphene-based coatings and clinical application of coated medical devices.

The in vitro laboratory testing is performed by means of single bacterial species, whereas multiple species’ biofilms are formed on the medical devices in the human body. The practical application of antimicrobial graphene materials and coatings requires additional testing in real environments, combined with a biological safety evaluation.

A number of characteristics of graphene materials and coatings are known, influencing their biological activity, such as chemical composition, particles size, sheets number, horizontal or vertical orientation, hydrophilic/hydrophobic balance, free energy, morphology and roughness of the coatings, etc. Control over these characteristics could be a tool to adjust the antimicrobial performance of the coated medical devices.

Different antimicrobial mechanisms of graphene materials are proposed, so far, based on physical and physicochemical interactions such as contact destruction, production of reactive oxygen species, etc., but the mechanisms of antimicrobial action of the graphene materials remain not fully understood.

## Figures and Tables

**Table 1:** Biologically active graphene (Gr) nanocomposites.

Graphene Nanocomposite	Preparation Mode	In Vitro Antibacterial Activity	**Ref.**
Gr/Ag NPs/iron NPs	Growth of Ag and iron NPs on the surface	*E. coli*; *S. aureus*	[37]
Gr/cadmium sulfide	Decoration	*E. coli* in presence of humic acid, under visible light	[38]
RGO/Ag NPs	Using hyaluronic acid template	*E. coli* in presence of humic acid, under visible light	[39]
GO/Hyaluronic acid/Ag NPs	Co-precipitation	Human pathogenic *S. aureus*; *E. coli*; *P. mirabilis*, *S. aureus* in vitro and wound disinfection model in vivo	[40]
RGO/Ag NPs (diam.16 ± 3.7 nm)	Sonication decoration	*S. aureus; E. coli, P. aeruginosa*	[41]
GO/Ag_2_O	In situ method; glucose as reducing agent	Multidrug resistant *E. coli*, *P. aeruginosa*; *K. pneumonia*; *S. aureus*	[42]
GO/Ag NPs	Without dispersing agent	*S. aureus*; *S. enterica*	[43]
RGO/Ag NPs	Film on dentin	*S. aureus*; *E. coli* *E. faecalis*	[44]
GO	Ultrasound assisted conditions	*S. aureus*	[45]
GO/Ag, water soluble	One step procedure, without surfactants and reductant	*E. coli*	[46]
GO/Ag	Exfoliation	*E. coli*	[47]
GO/Ag NPs	Co-precipitation; green reducing agent	*E. coli*	[48]
RGO/copper	Decoration	Algicidal activity without toxicity to mammalian cells	[49]
RGO/Ag	Modified method of Hummer	*E. coli*	[50]
RGO/Ag/Cu	Modified method of Hummer	*E. coli*	[51]
RGO/ZnO/TiO_2_/SiO_2_	Modified method of Hammer	*E. coli*	[52]

**Table 2:** Antimicrobial coatings based on graphene, graphene derivatives, and graphene nanocomposites.

Coating	Deposition Mode	Potential Application	Ref.
**Gr, GO and RGO coatings**			
Graphene coating, horizontally grown	Chemical vapor deposition (CVD)	A variety of applications	[76]
Graphene coating, vertically grown	Plasma-enhanced CVD	A variety of applications	[77]
Gr NPs coating	Dip and spray coating	Silicone rubber catheters	[78]
Atom thick GR coating	Hot pressing, dry transfer	Medical grade titanium	[79]
Gr coating from recycled pencil tubes	Sonication exfoliation	Protection against pathogenic bacteria	[80]
GO coating	Spraying	Antibiofilm protection	[81]
Uniform Gr film	Ultrafast CVD	A variety of applications	[82]
Bio-RGO coating	Spaying	Medical devices	[83]
Gr coatings with tunable wettability	Surface immobilization	Smart material surfaces	[65]
Surfaces functionalized by 2D GO	Poly(dopamine) chemistry	Water purification membranes	[74]
Small, oxidized GR nanoplatelets on polyurethane (PU)	Meld blending and dip coating	Antimicrobial protection of PU	[84]
Vertically and horizontally aligned Gr on semiconductor silicone (Si) and insulator silicon dioxide (SiO_2_)	CVD	Antibiofouling protection	[85]
Coal derived GO coatings	One pot process	Titanium implants	[86]
**Gr nanocomposite coatings**			
Porous polyelectrolyte coating with RGO flakes	Layer-by-layer deposition	Cardiovascular devices	[87]
Porous silver/hydroxyapatite/graphene coating	Electrophoretical deposition	Medical grade titanium	[88]
Gr- and Ag-NPs decorated GR nanolayers	Spray coating	A variety of applications	[89]
Chitosan cross-linked GO nanocomposite coating	Spraying	A variety of applications	[73]
A ternary Si/GO/Al_2_O_3_ hybrid nanorods coatings	Solution casting	Superhydrophobic antifouling coatings	[90]
GO based nanocomposites, decorated with Ag NPs	Ultrasonic deposition	Multifunctional antibacterial and antifungal protection of SiO_2_/Si substrates	[91]
RGO/TiO_2_ nanocomposite coating	Deeping	Cotton fabrics	[92]
Gr/silicon rubber	Spray coating	Biofouling prevention	[93]
GO and lysozyme ultrathin films	Surface immobilization	Antibacterial protection and improved ontogenesis (orthopedic)	[94]
Chitozan hybrid films/N-halamin-functionalized GO	Surface immobilization	Medical devices	[95]
Hydroxyapatite/GO/ZrO composite coating	Electrophoretic deposition	Titanium substrates	[95]
GO/Si/PDMS coating	Spraying, brushing	Carbon steel anticorrosion and antimicrobial protection	[96]
Hybrid PDMS/Epoxy/Worm-like GO nanoscrolls	Multistep procedure	AA24 alloy anticorrosion and antimicrobial protection	[97]
Modified GO/PDMS	Multistep procedure	Hydrophobic antifouling surfaces	[98]
Hybrid GO/Ag NPs nanocomposite coatings	Multistep procedure	Antibiofilm applications	[60]
